# Synthesis of organic–inorganic hybrid nanocomposites modified by catalase-like catalytic sites for the controlling of kiwifruit bacterial canker†

**DOI:** 10.1039/d4ra02006e

**Published:** 2024-05-31

**Authors:** Zhenghao Ding, Qingqing Song, Guangdi Wang, Zhuojun Zhong, Guoyong Zhong, Hong Li, Yuexin Chen, Xiang Zhou, Liwei Liu, Song Yang

**Affiliations:** a State Key Laboratory of Green Pesticide, Key Laboratory of Green Pesticide and Agricultural Bioengineering, Ministry of Education, Center for R&D of Fine Chemicals of Guizhou University Guiyang 550025 China syang@gzu.edu.cn jhzx.msm@gmail.com

## Abstract

Kiwifruit bacterial canker, caused by *Pseudomonas syringae* pv. *Actinidiae* (*Psa*), is one of the most important diseases in kiwifruit, creating huge economic losses to kiwifruit-growing countries around the world. Metal-based nanomaterials offer a promising alternative strategy to combat plant diseases induced by bacterial infection. However, it is still challenging to design highly active nanomaterials for controlling kiwifruit bacterial canker. Here, a novel multifunctional nanocomposite (ZnO@PDA-Mn) is designed that integrates the antibacterial activity of zinc oxide nanoparticles (ZnO NPs) with the plant reactive oxygen species scavenging ability of catalase (CAT) enzyme-like active sites through introducing manganese modified polydopamine (PDA) coating. The results reveal that ZnO@PDA-Mn nanocomposites can efficiently catalyze the conversion of H_2_O_2_ to O_2_ and H_2_O to achieve excellent CAT-like activity. *In vitro* experiments demonstrate that ZnO@PDA-Mn nanocomposites maintain the antibacterial activity of ZnO NPs and induce significant damage to bacterial cell membranes. Importantly, ZnO@PDA-Mn nanocomposites display outstanding curative and protective efficiencies of 47.7% and 53.8% at a dose of 200 μg mL^−1^ against *Psa in vivo*, which are superior to those of zinc thiozole (20.6% and 8.8%) and ZnO (38.7% and 33.8%). The nanocomposites offer improved *in vivo* control efficacy through direct bactericidal effects and decreasing oxidative damage in plants induced by bacterial infection. Our research underscores the potential of nanocomposites containing CAT-like active sites in plant protection, offering a promising strategy for sustainable disease management in agriculture.

## Abstract 2

The catalase-like catalytic activity of the ZnO@PDA-Mn nanocomposites reduced the oxidative damage of plants induced by bacterial infection, and effectively enhanced the control effect of kiwifruit canker disease.

## Graphical abstract

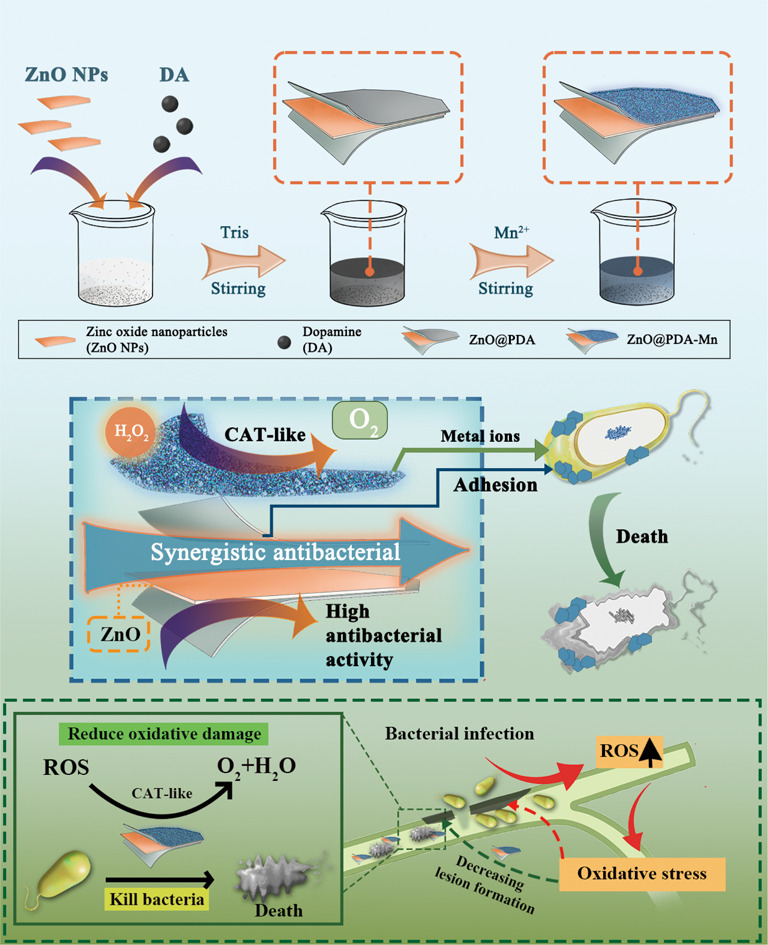

## 1. Introduction

The kiwifruit is a globally and widely cultivated fruit for its significant economic and nutritional value.^1^ However, bacterial canker disease caused by *Pseudomonas syringae* pv. *Actinidiae* (*Psa*) has been one of the most serious diseases in kiwifruits, which has significantly reduced the yield and quality of kiwifruits. *Psa* can be spread by wind and infect tissue through wounds, then manifests in vine and trunk ulcers, soft stems, and rattan wilt, ultimately leading to plant demise.^2,3^ Kiwifruit canker disease has been spreading over large areas in major kiwifruit-growing countries including China, New Zealand, Italy, Korea, Iran, France, Switzerland, and Australia, and leading to severe economic losses worldwide.^4^ For instance, kiwifruit canker disease caused an estimated cost of export losses alone increasing to NZ$930 million in damage to the New Zealand economy for the four years 2011–2014.^5^ Unfortunately, there is no efficient curative treatment method known for *Psa*.^6^ Current strategies for combating kiwifruit bacterial canker primarily rely on preventive methods involving breeding resistant varieties, and chemical or biological agent control methods.^7,8^ However, the time-consuming nature of breeding resistant cultivars and the inherent instability of biological control effectively underscore the continued reliance on chemical interventions.^8,9^ Moreover, the effectiveness of traditional bactericides against *Psa* is limited by its Gram-negative structure, which boasts an effective permeability barrier, rendering many treatments ineffective.^10,11^ Once the disease breaks out, there are few appropriate ways to control its spread.^12–15^ Therefore, there is a growing need to develop novel chemical agents for targeting kiwifruit canker.

Currently, nanotechnology has become one of the most effective technologies for conquering infectious diseases in humans or plants.^16–18^ In particular, metals and metal oxide nanoparticles possess special physical and chemical properties due to their small size and large specific surface area, which can kill bacteria in a variety of ways, including special particle size effects, strong surface interactions, and the release of metal ions.^19–22^ Recent advancements in nanomaterials have showcased their great potential in pest and crop disease management due to their potent biological activity.^23^ Metal and metal oxide nanoparticles, such as silver (Ag), zinc (Zn), zinc oxide (ZnO), manganese oxide (MnO), and iron oxide (Fe_*x*_O_*y*_), have exhibited remarkable antibacterial properties, making them promising candidates for plant protection.^24–28^ For example, Awasthi *et al.* have demonstrated that green synthetic ZnO nanoparticles exhibit potent antibacterial and antibiofilm activity against *Bacillus subtilis*, suggesting their potential as a therapeutic alternative for biofilm-producing and drug-resistant bacteria.^29^ Furthermore, Li *et al.* successfully synthesized environmentally friendly ZnO, manganese dioxide (MnO_2_), and magnesium oxide (MgO) nanoparticles, all of which exhibited significant bacteriostatic effects on rice bacterial leaf blight disease and contributed to the improvement of rice growth parameters and biomass.^30^ However, there's a scarcity of nanoparticles tailored specifically for combating kiwifruit canker disease.^31^ Our previous studies have demonstrated the enhanced control of kiwifruit bacterial canker *in vitro* using nanoparticles containing zinc or copper.^32,33^ Nanoparticles with simple and single functions usually exhibit superior antibacterial activity *in vitro*, but the bactericidal activity *in vivo* is not significantly improved. Whereas, composite nanomaterials by reasonable designed can improve the antibacterial effect, systemic effect in plant and biocompatibility, so as to improve the protective activity and curative activity of plant diseases *in vivo*.^34–36^ Hence, proposing a novel design strategy of composite nanomaterials for the effective prevention and control of kiwifruit canker holds immense promise for managing plant diseases.

Among all antimicrobial nanomaterials, nanozymes, that is, artificial nanomaterials with the catalytic effect of biological enzymes, have become one of the most important newly-developing materials in the field of treating bacterial infectious diseases.^37–40^ Due to their unique characteristics, such as high activity, stability, tunable activity, and multifunctionality for scaling up, nanozymes often have more complex biological functions in comparison to enzymes, traditional enzyme mimics, and common nanomaterials.^41^ The peroxidase and oxidase-like nanozymes have been demonstrated to catalyze producing harmful reactive oxygen species (ROS), which can serve as bactericidal agents, but also pose a potential risk of damaging healthy tissue cells.^42^ In addition, nanozymes, as important antioxidants, have been extensively utilized in mitigating oxidative damage caused by abiotic stress.^43^ Among these nanozymes, catalase (CAT)-like nanozymes mimic the activity of catalase, catalyzing the decomposition of hydrogen peroxide (H_2_O_2_) into water (H_2_O) and oxygen (O_2_).^44^ For instance, manganese oxide (Mn_3_O_4_) nanoparticles, acting as CAT-like nanozymes, promote cucumber plant growth by alleviating oxidative stress.^45^ Additionally, cerium oxide (CeO_2_) nanoparticles protect plant photosynthesis from abiotic stresses by removing reactive oxygen species.^46^ Compared with abiotic stress, bacterial infection subjected crops to multiple stress from bacteria and plant immune responses. When plants are affected by abiotic stress and biological stress, effectors induce plant immune responses, leading to the generation of ROS, such as H_2_O_2_, exacerbating disease symptoms.^47^ In addition, accumulating evidence suggests that ROS accumulation promotes bacterial infection, and the inability to maintain ROS homeostasis may lead to plant chlorosis and necrosis.^48^ Therefore, at the same time the nanomaterials kill the bacteria, the timely removal of endogenous reactive oxygen species is of great significance for the prevention and control of bacterial diseases in plants. However, there are few reports on nanozymes in biological stress. Additionally, nanomaterials with CAT-like activity containing individual transition metals usually have poor bactericidal activity, which may be insufficient for the effective prevention and control of plant bacterial diseases.^49^ To address these limitations, it has important implications for controlling kiwifruit canker disease to develop novel multifunctional nanocomposites with both high antibacterial activity and ROS scavenging effect.

**Scheme 1:**
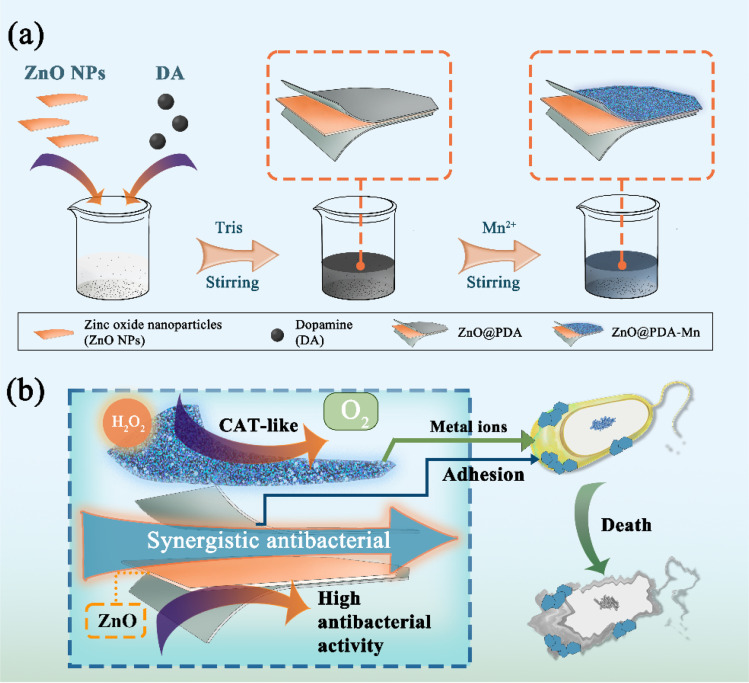
(a) Illustration for the synthesis of ZnO@PDA-Mn nanocomposite. (b) Synergistic antibacterial mechanism of ZnO@PDA-Mn.

Herein, we present the design and synthesis of a novel multifunctional hybrid nanocomposite (ZnO@PDA-Mn) tailored to target kiwifruit bacterial canker disease. Through a simple one-pot, two-step process, polydopamine (PDA) modified ZnO nanoparticles (ZnO NPs) are decorated with catalase-mimic Mn(ii), forming an effective strategy for controlling kiwifruit canker disease (Scheme 1). First, low-cost commercial ZnO NPs serve as the active core of nanocomposite against *Psa*.^50–54^ In addition, the incorporation of PDA ensures uniform deposition of the metal enzyme-like active sites on the surface, enhancing biocompatibility.^55^ The nanocomposites, with excellent CAT-like activity, exhibit *K*_m_ and *V*_ma*x*_ values superior to that of most reported nanozymes, efficiently converting H_2_O_2_ into O_2_. Due to the antibacterial properties of ZnO and the adhesion of PDA, ZnO@PDA-Mn exhibits superior antibacterial activity compared to zinc thiozole *in vitro*, achieving a 100% bactericidal rate against *Psa* at 1.57 μg mL^−1^. Interestingly, ZnO@PDA-Mn significantly enhanced *in vivo* activity compared to the control drug, attributed to its potent antibacterial efficacy and capability to mitigate plant ROS levels. Overall, the ZnO@PDA-Mn nanocomposites offer promising prospects for precision control of agricultural bacterial diseases and highlight the potential of nanozymes in plant protection strategies.

## 2. Materials and methods

### 2.1. Materials

All chemicals were of analytical grade. Dopamine hydrochloride, 98%; zinc oxide nanoparticles, 99.8% metals basis, 50 ± 10 nm; manganese sulfate monohydrate, AR; 3,3′,5,5′-tetramethylbenzidine (TMB) were purchased from Energy Chemical. H_2_O_2_ (30%) were purchased from Tianjin ZhiYuan Reagent Co., Ltd.

### 2.2. Synthesis of ZnO@PDA-Mn nanocomposites

ZnO@PDA-Mn nanocomposites was synthesized by a one-pot strategy. Briefly, 5 mg of ZnO nanoparticles were dissolved in dd H_2_O (10 mL), dispersed by ultrasound for 10 minutes (30 W). Add 80 μL DA (10 mg mL^−1^) to the mixture and 80 μL Tris(hydroxymethyl)aminomethane (Tris) buffer (6 mg mL^−1^), stirring at room temperature for 500 rpm overnight to get ZnO@PDA solution. After that, 1 mL of MnSO_4_ (1.3 mg mL^−1^) was further added to the mixture solution and the pH value is adjusted to 8 with Tris. The reaction was then performed for 24 hours until the solution turned brown. The precipitate was obtained by centrifugation (10 000 rpm, 5 min), washed 3 times with dd H_2_O and 3 times with absolute ethanol, and the product was naturally dried.

### 2.3. Catalase-like activity of nanocomposites

The CAT-like activity of the ZnO@PDA-Mn nanocomposites was evaluated at room temperature by measuring generated O_2_ solubility (unit: mg L^−1^) at different reaction times using a specific oxygen electrode on a Multi-Parameter Analyzer (JPSJ-606L, Leici, China). The kinetics assays of the ZnO@PDA-Mn (final concentration 100 μg mL^−1^) with H_2_O_2_ as the substrate were performed by adding different amounts of H_2_O_2_ (135 μL of 7.5%, 125 μL of 15%, and 135, 270, 1080, and 2160 μL of 30% H_2_O_2_) solution to 18 mL of phosphate buffer saline solution (PBS, pH = 7). For comparison, the enzyme-like activities of ZnO NPs and ZnO@PDA were also measured.

### 2.4. Antibacterial effect of nanocomposites

The antibacterial efficacy of the ZnO@PDA-Mn nanocomposites against bacteria was evaluated by quantifying the number of colony-forming units (CFU) employing the plate counting method. Ten groups of bacteria were tested: (I) bacteria; (II) bacteria + H_2_O_2_ (1 mM); (III) bacteria + thiazole zinc (3.125 μg mL^−1^); (IV) bacteria + ZnO (3.125 μg mL^−1^); (V) bacteria + ZnO@PDA-Mn (3.125 μg mL^−1^); (VI) bacteria + ZnO@PDA-Mn (3.125 μg mL^−1^) + H_2_O_2_ (1 mM); (VII) bacteria + thiazole zinc (1.57 μg mL^−1^); (VIII) bacteria + ZnO (1.57 μg mL^−1^); (IX) bacteria + ZnO@PDA-Mn (1.57 μg mL^−1^); (X) bacteria + ZnO@PDA-Mn (1.57 μg mL^−1^) + H_2_O_2_ (1 mM). In brief, *Psa* were cultured overnight in LB medium at 28 °C in an incubator shaker. Subsequently, the bacteria (OD = 0.6–0.8) were diluted 10-fold, and 100 μL of the diluted bacteria were added to 1.5 mL centrifuge tubes. The final bacterial concentration was 1.0 × 10^6^–1.0 × 10^7^ CFU per mL in PBS buffer. After incubating the mixed suspension at room temperature for 8 hours, it was diluted 100-fold, and 20 μL was evenly spread on solid medium to determine CFU.

### 2.5. Morphological observation of bacteria

Following the assessment of antibacterial efficacy, bacterial suspensions underwent a 4 hour treatment with I–X. Subsequently, these suspensions underwent centrifugation, followed by re-dispersion in 2.5% glutaraldehyde solution (Sigma-Aldrich, USA) for 4 hours at 4 °C in darkness. The bacterial cells were subsequently dehydrated successively with ethanol concentrations of 30%, 50%, 70%, 90%, and finally 100%, each for a duration of 10 minutes. Ultimately, the dehydrated bacteria were coated with gold and imaged using scanning electron microscopy.

### 2.6. *In vivo* antibacterial bioassay targeting kiwifruit bacterial canker

Our previously established method was employed to assess the *in vivo* antibacterial effects of samples against kiwifruit bacterial canker.^32^ Zinc thiozole was utilized as the positive control. A 2 year-old kiwifruit plant of the Hong Yang Hongxin variety was chosen for the trial. Each kiwifruit tree underwent the creation of three incisions, approximately 0.1 cm wide, extending to the phloem. In the protective test, 10 μL of either nanoparticles or zinc thiozole solution at a concentration of 200 μg mL^−1^ was applied to the wounds. After a 24 hour interval, 10 μL of *Psa* (OD_595_ = 0.2) was introduced into all incisions. For the curative test, 10 μL of *Psa* suspension (OD_595_ = 0.2) was initially introduced into all wounds, followed by the administration of 10 μL of nanoparticles or zinc thiozole solution at 200 μg mL^−1^ after 24 hours. Subsequently, all treatment groups were cultivated in a climate chamber under conditions comprising 14 hours of light at 16 °C and 10 hours of darkness at 10 °C, with 80% relative humidity. The curative and protective effects were then observed and quantified 14 days post-inoculation.

### 2.7. Statistical analysis

For all statistical data, the number of independent experiments was denoted as *n*. Statistical analysis was conducted using SPSS Statistics (version 22.0, IBM) employing one-way analysis of variance (ANOVA) with a Tukey *post hoc* approach.

## 3. Results and discussion

### 3.1. Synthesis and characterization

In this study, the ZnO@PDA-Mn nanocomposites were synthesized using a simple two-step one-pot method. Initially, commercial ZnO nanoparticles served as the active core, while polydopamine (PDA) was employed as the coating material. Subsequently, Mn(ii) was added after the formation of ZnO@PDA.

**Fig. 1:**
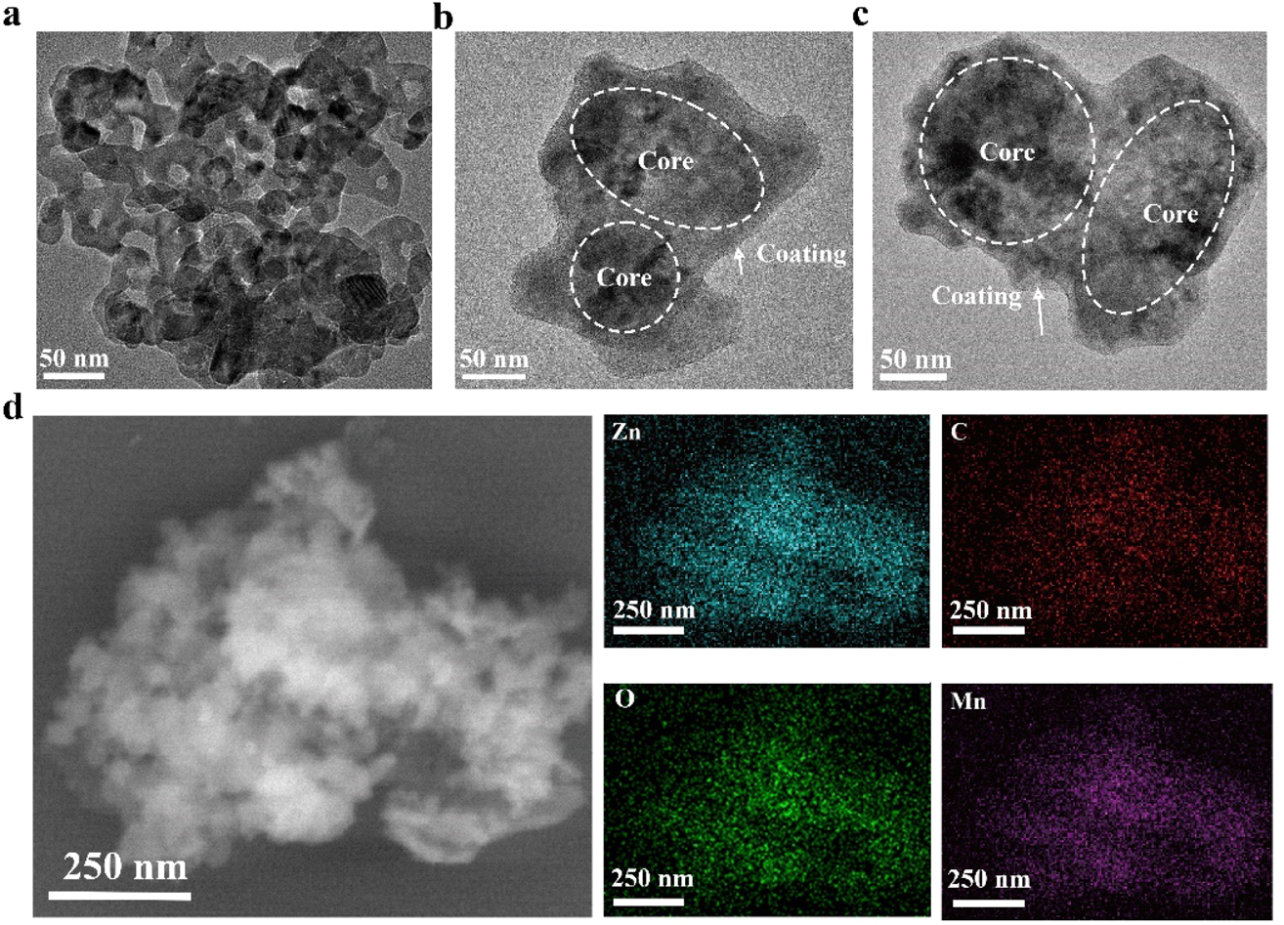
TEM images of (a) ZnO nanoparticles, (b) ZnO@PDA nanocomposites and (c) ZnO@PDA-Mn nanocomposites. (d) Scanning electron microscopy (SEM) images and energy dispersive spectroscopy (EDS) mapping of ZnO@PDA-Mn nanocomposites (dotted circles highlight the presence of the nanocomposites core).

Transmission electron microscopy (TEM) characterization (Fig. 1a–c) revealed that both the synthesized ZnO@PDA and ZnO@PDA-Mn nanocomposites exhibited a core–shell like structure with a polymer coating, which are different from ZnO NPs. This core–shell like structure is consistent with previous reports on nanomaterials coated with PDA, confirming the successful attachment of PDA coatings.^56^ Furthermore, element mapping (Fig. 1d) of Zn, C, O, and Mn in ZnO@PDA-Mn provided further insights into its composition.

**Fig. 2:**
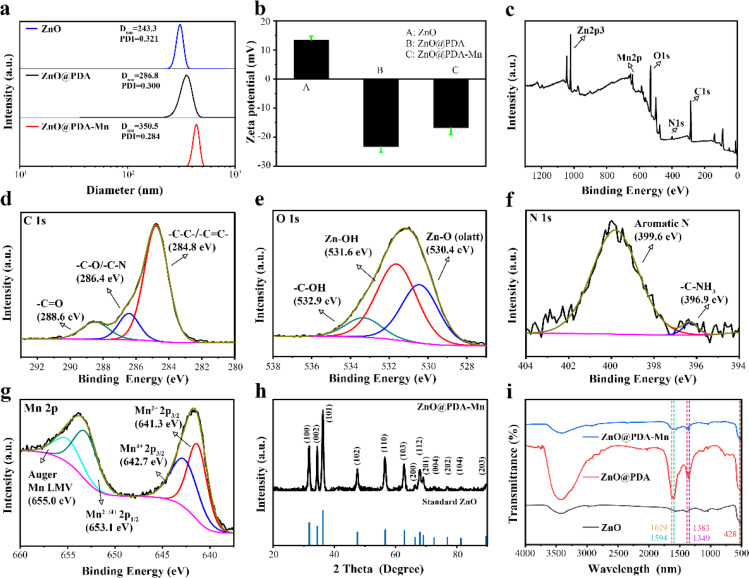
(a) Dynamic light scattering (DLS) analysis of ZnO, ZnO@PDA and ZnO@PDA-Mn. (b) Zeta-potentials of ZnO, ZnO@PDA and ZnO@PDA-Mn. (c) XPS full scan spectrum ZnO@PDA-Mn. XPS high-resolution scans of C 1s (d), O 1s (e), N 1s (f), and Mn 2p (g) of ZnO@PDA-Mn. (h) XRD patterns of ZnO@PDA-Mn. (i) FT-IR spectra of ZnO, ZnO@PDA and ZnO@PDA-Mn.

The ZnO@PDA-Mn nanocomposites displayed outstanding water dispersibility. As shown in Fig. 2a, the size of the ZnO@PDA-Mn nanocomposites were 350.5 nm measured by dynamic light scattering (DLS), which is slightly larger than ZnO and ZnO@PDA, probably due to the continuous polymerization of PDA. The zeta potential of the ZnO was 13.4 mV and eventually regulated at −16.8 mV after PDA adhesion and Mn(ii) decoration (Fig. 2b). X-Ray photoelectron spectroscopy (XPS) analysis (Fig. 2c–g) provided insights into the elemental composition and chemical states of ZnO@PDA-Mn. As shown in Fig. 2c, the ZnO@PDA-Mn were mainly composed of C (55.23 atom%), O (31.62 atom%), Zn (6.37 atom%), N (3.52 atom%) and Mn (3.25 atom%), which indicates the successful preparation of the ZnO@PDA-Mn. The energy dispersive spectrometer (EDS) indicates that the mass fraction of Mn in ZnO@PDA-Mn is about 10.22%, which indicates that Mn is successfully attached to the surface (Fig. S2†). The high-resolution Mn 2p spectrum (Fig. 2g) has four main peaks, and the peaks at 641.3 eV and 642.7 eV are assigned to the Mn 2p_3/2_ features of Mn^2+^ and Mn^4+^, the peaks at 653.1 eV are attributed to Mn 2p_1/2_ features of Mn^2+^/Mn^4+^, which provide the structural basis for the enzymatic reactions.^57–59^ High-resolution XPS spectra of C 1s, N 1s and O 1s furtherly confirmed successful PDA loading onto ZnO NPs. The XRD pattern of ZnO@PDA-Mn displayed that all the diffraction peaks were consistent with the ZnO crystal, which proves that Mn ions may coordinate on the PDA coating (Fig. 2h).

The Fourier transform infrared (FT-IR) spectrometer was utilized to analyze the functional groups of ZnO@PDA-Mn nanocomposites, with the resulting spectra displayed in Fig. 2i. The band centered at 450 cm^−1^ was assigned to the Zn–O stretching vibration. In addition, the peak observed at 1629 cm^−1^ corresponds to the stretching vibration of aromatic rings within the PDA backbone, while the absorption peak at 1594 cm^−1^ is attributed to the scissoring vibration of the N–H bond. Furthermore, bands at 1349 and 1383 cm^−1^ are associated with the stretching and bending vibration of C–O groups on the benzene ring, respectively. These distinctive peaks at 1629, 1594, 1383, and 1349 cm^−1^ serve as confirmatory evidence for the polymerization of PDA.^60^ The above observations confirmed that the ZnO@PDA-Mn structure was successfully achieved and efficiently decorated with PDA and Mn.

### 3.2. Extracellular enzyme-like activity

**Fig. 3:**
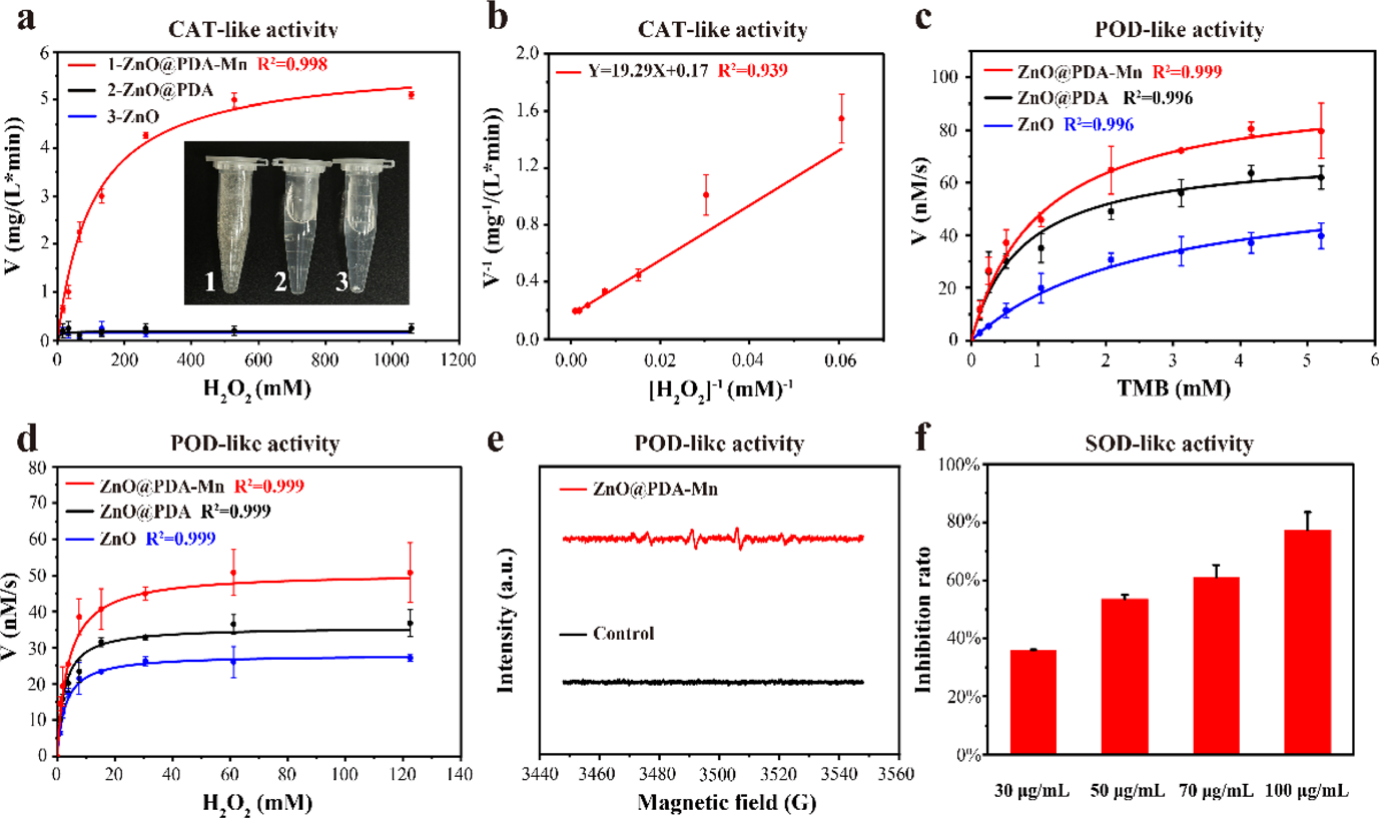
(a) Steady-state kinetics assay of CAT-like activity of ZnO@PDA-Mn with varied H_2_O_2_. (b) Lineweaver–Burk plotting of CAT-like activity for ZnO@PDA-Mn with H_2_O_2_ as a substrate at room temperature (25 °C). (c) Evaluation of the steady-state kinetics assay for the POD-like activity of ZnO@PDA-Mn using varying concentrations of TMB. (d) Assessment of the steady-state kinetics assay for the POD-like activity of ZnO@PDA-Mn using varying concentrations of H_2_O_2_. (e) Analysis of electron spin resonance (ESR) spectra depicting hydroxyl radicals produced by ZnO@PDA-Mn in the presence of H_2_O_2_. (f) Detection of SOD-like activity of ZnO@PDA-Mn through WST-1 assay.

Following the successful construction of ZnO@PDA-Mn, its enzyme-like catalytic ability was investigated. Firstly, we examined its O_2_ generation ability (CAT-like activity) at a concentration of 100 μg mL^−1^ in varying amounts of H_2_O_2_. The kinetic data were obtained by monitoring the O_2_ generation in the catalytic reaction system within 1 minute, in which the higher the concentration of H_2_O_2_, the more O_2_ was generated. Subsequently, the reaction rate was fitted against the corresponding H_2_O_2_ concentrations to obtain Michaelis–Menten curves (Fig. 3a). The Michaelis–Menten constants (*K*_m_) and the maximum velocity (*V*_ma*x*_) were determined through linear double-reciprocal plot (Linearweave–Burke fitting) (Fig. 3b). Based on these calculations, the *K*_m_ of ZnO@PDA-Mn nanocomposites against H_2_O_2_ were determined to be 116.3 mM, while the *V*_ma*x*_ was 6.03 mg (L min)^−1^. These values are consistent with previously reported catalase-like nanozymes (Table S1†), indicating that ZnO@PDA-Mn nanocomposites possess competent catalase mimicry abilities, capable of rapidly reducing ROS accumulation in plants.^61–65^

Furthermore, we evaluated other enzyme-like activities of ZnO@PDA-Mn nanocomposites, including peroxidase (POD)-like activity and superoxide dismutase (SOD)-like activity. The assessment of POD-like activity involved the oxidation of 3,3′,5,5′-tetramethylbenzidine (TMB), with ZnO, ZnO@PDA and PDA employed as control groups. The kinetic assay of POD-like activity was examined by monitoring dynamic variations in absorbance at 652 nm, using TMB and H_2_O_2_ as substrates (Fig. 3c, d and S3†). ZnO@PDA-Mn exhibited relatively high POD-like activity compared to ZnO, ZnO@PDA and PDA, capable of inducing H_2_O_2_ conversion to ·OH under weak acid conditions (pH = 4.5). Additionally, Electron Spin Resonance (ESR) spectra confirmed that ZnO@PDA-Mn catalyzed H_2_O_2_ to form ˙OH (Fig. 3e). In addition to CAT and POD-like activities, ZnO@PDA-Mn nanocomposites exhibited SOD-like activity (Fig. 3f). At concentrations of 30, 50, 70, and 100 μg mL^−1^, the O_2_˙^−^ scavenging rate of ZnO@PDA-Mn detected by WST-1 was measured at 35.8%, 53.6%, 61.0%, and 77.4%, respectively. The multienzyme-like properties of ZnO@PDA-Mn nanocomposites may provide valuable insights into the biological effects of Mn-based nanomaterials under plant physiological conditions.

### 3.3. Antibacterial activity *in vitro*

**Fig. 4:**
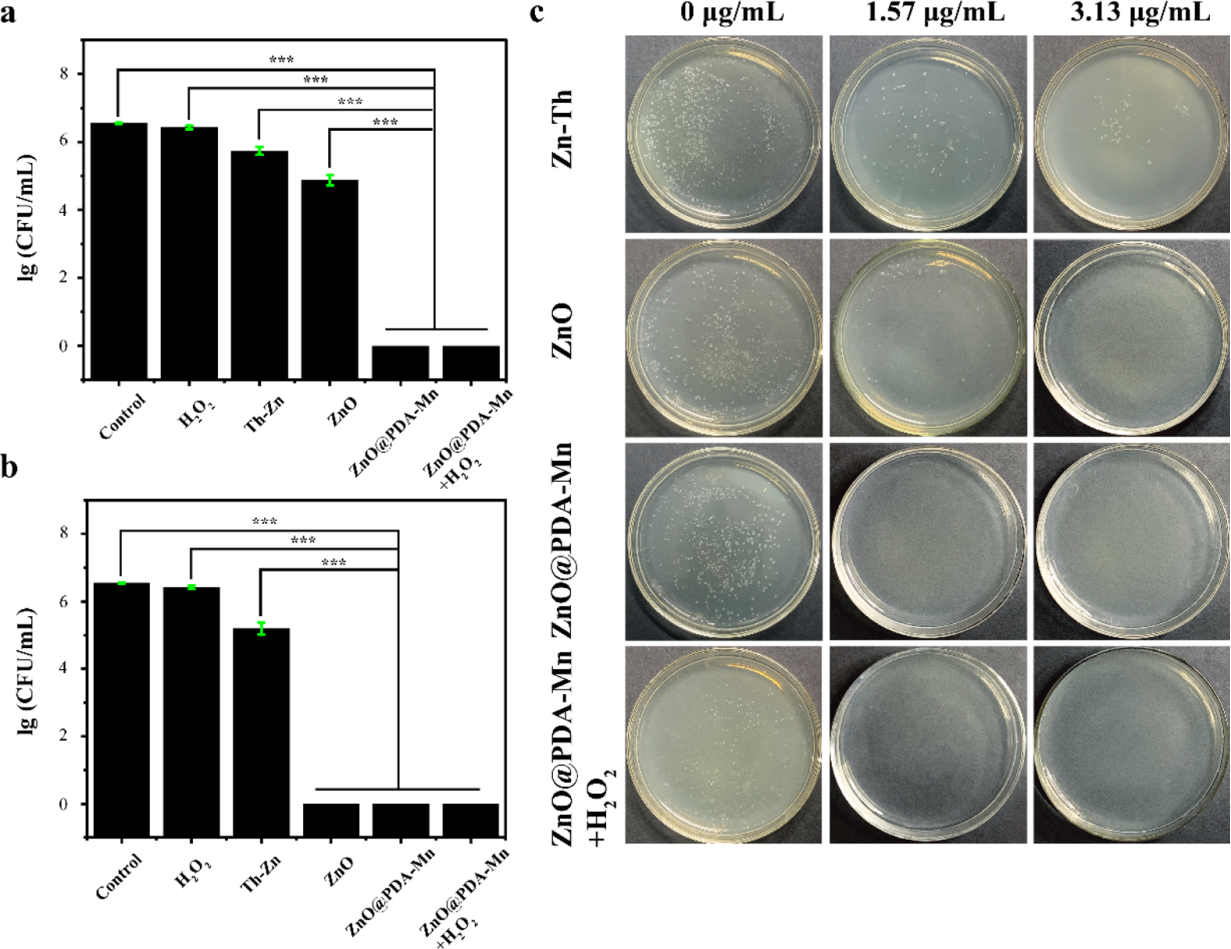
(a) Antibacterial activity of H_2_O_2_, Zn–Th, ZnO, ZnO@PDA-Mn and ZnO@PDA-Mn + H_2_O_2_ against *Psa*. Concentrations of Zn–Th, ZnO, and ZnO@PDA-Mn were 1.57 μg mL^−1^ H_2_O_2_ (1 mM). (b) Antibacterial activity of H_2_O_2_, Zn–Th, ZnO, ZnO@PDA-Mn and ZnO@PDA-Mn + H_2_O_2_ against *Psa*. Concentrations of Zn–Th, ZnO, and ZnO@PDA-Mn were 3.125 μg mL^−1^ H_2_O_2_ (1 mM). (c) Visualization of representative bacterial colony formation across various treatment groups. Significance analysis was carried out by one-way ANOVA and *t* test. Single asterisks indicate *p* < 0.05, double asterisks indicate *p* < 0.01, triple asterisks indicate *P* < 0.001.

The antibacterial activity of ZnO@PDA-Mn nanocomposites against *Psa* was evaluated using the viable cell counting method. Results for the antibacterial activities of zinc thiozole (Zn–Th), ZnO NPs, and ZnO@PDA-Mn nanocomposites are illustrated in Fig. 4. Remarkably, ZnO@PDA-Mn exhibited excellent antibacterial activity at a concentration of only 1.57 μg mL^−1^, with nearly 100% of *Psa* killed after exposure for 8 hours. Therefore, the minimum bactericidal concentration (MBC) of ZnO@PDA-Mn was evaluated to be 1.57 μg mL^−1^ (Fig. 4a).

Furthermore, ZnO NPs demonstrated significant antibacterial activity, indicating that ZnO serves as the primary antibacterial source of ZnO@PDA-Mn under neutral conditions. However, the antibacterial activity of ZnO@PDA-Mn was superior to that of ZnO NPs, possibly due to the modification of Mn ions.^66,67^ Additionally, the bactericidal effect of H_2_O_2_ on *Psa* was investigated, revealing no significant antibacterial effect on *Psa* at low concentrations of H_2_O_2_. Moreover, in the presence of H_2_O_2_, the antibacterial activity of ZnO@PDA-Mn did not significantly decrease. In addition, the antibacterial activity of ZnO@PDA-Mn nanocomposites were further evaluated by using the zone of inhibition method (Fig. S4†). The results showed that the zone of inhibition of Zn–Th, ZnO, ZnO@PDA-Mn and ZnO@PDA-Mn + H_2_O_2_ were 6.1 mm,6.8 mm, 8.2 mm and 7.8 mm, respectively at a concentration of 3.13 μg mL^−1^. It also proved that Zn–Th, ZnO, ZnO@PDA-Mn had antibacterial activity against *Psa*, wherein ZnO@PDA-Mn had better antibacterial activity compared to other controls. In addition, the antibacterial activity of ZnO@PDA-Mn does not decrease significantly after the conversion of H_2_O_2_ to O_2_ and H_2_O. This experimental result is similar to the CFU data. Concerning *Xanthomonas axonopodis* pv. *citr* (*Xac*), at a concentration of 1.57 μg mL^−1^, no bacterial colonies were observed in the ZnO@PDA-Mn group (Fig. S5†), indicating superior antibacterial activity compared to commercial Zn–Th *in vitro*.

### 3.4. Antibacterial mechanism

**Fig. 5:**
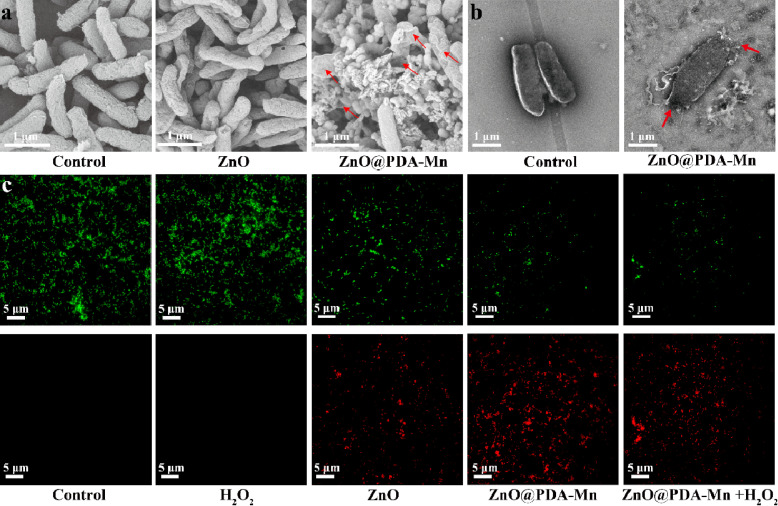
(a) SEM images of *Psa* after treatment with Control (PBS), ZnO, ZnO@PDA-Mn. (b) TEM images of *Psa* after treatment with Control (PBS), ZnO, ZnO@PDA-Mn. (c) Live/dead bacterial viability assessment of *Psa* (the red arrows indicate that the bacteria were significantly deformed after treatment with ZnO@PDA-Mn).

Scanning electron microscopy (SEM) was employed to evaluate the antibacterial mechanism of the ZnO@PDA-Mn nanocomposites by examining morphological changes and membrane damage of bacteria under various treatments, as depicted in Fig. 5a. Bacteria treated with PBS maintained a typical rod shape with an intact surface. In contrast, bacterial (*Psa*) treated with ZnO@PDA-Mn nanocomposites exhibited severe deformation of the bacterial membrane, characterized by shrinkage and rupture of the cell membrane leading to cytoplasmic outflow. However, the effect of the same concentration of ZnO NPs was relatively small, with only partial damage to the bacteria. SEM images revealed strong interaction between ZnO@PDA-Mn and bacteria, possibly due to PDA adhesion.^68^ This strong interaction may be one of the main reasons for the significant deformation of the bacterial membrane. Additionally, the attachment of Mn ions may also contribute to damaging the bacterial cell membrane. TEM images further showed that after ZnO@PDA-Mn treatment, cell membrane rupture resulted in cytoplasmic outflow (Fig. 5b), consistent with what the SEM images indicate. Therefore, ZnO@PDA-Mn nanocomposites effectively destroy the structure of the bacterial membrane to efficiently kill bacteria.

To further verify the bactericidal effect of ZnO@PDA-Mn against *Psa* bacteria, STYO 9 and propidium iodide (PI) was used as a fluorescent probe to detect changes in cell membrane permeability. STYO 9 can label bacteria to emit green fluorescence, while PI can enter cells and bind with DNA, emitting red fluorescence when the membrane permeability of bacteria changes (Fig. 5c). The control group and the H_2_O_2_-treated bacteria showed no signs of red fluorescence, indicating that H_2_O_2_ had little effect on the permeability of *Psa* cell membranes. Bacteria treated with ZnO NPs and ZnO@PDA-Mn nanocomposites exhibited obvious red fluorescence signal, confirming the mechanism of their action on the cell membrane.^69,70^ Importantly, compared with ZnO@PDA-Mn, there was no significant change in the red fluorescence signal of bacteria treated with ZnO@PDA-Mn nanocomposites and low concentration of H_2_O_2_ (1 mM), indicating that the antibacterial activity of ZnO@PDA-Mn did not decrease significantly after its catalytic conversion of H_2_O_2_ to O_2_ and H_2_O. While previous studies have reported that nanozymes with CAT-like activity can exhibit antibacterial effects and disrupt biofilms through bubble dynamics, no similar phenomenon was observed in this study.^71^ This discrepancy may be attributed to the fact that the bacteria studied here, specifically *Psa* bacteria, are not anaerobic bacteria.

In summary, *in vitro* experiments indicate that ZnO@PDA-Mn nanocomposites possess a dual bactericidal mechanism involving active nanoparticles and strong interactions, and their antibacterial ability is not diminished in a low concentration of H_2_O_2_ environment.

### 3.5. Antibacterial activity *in vivo*

**Fig. 6:**
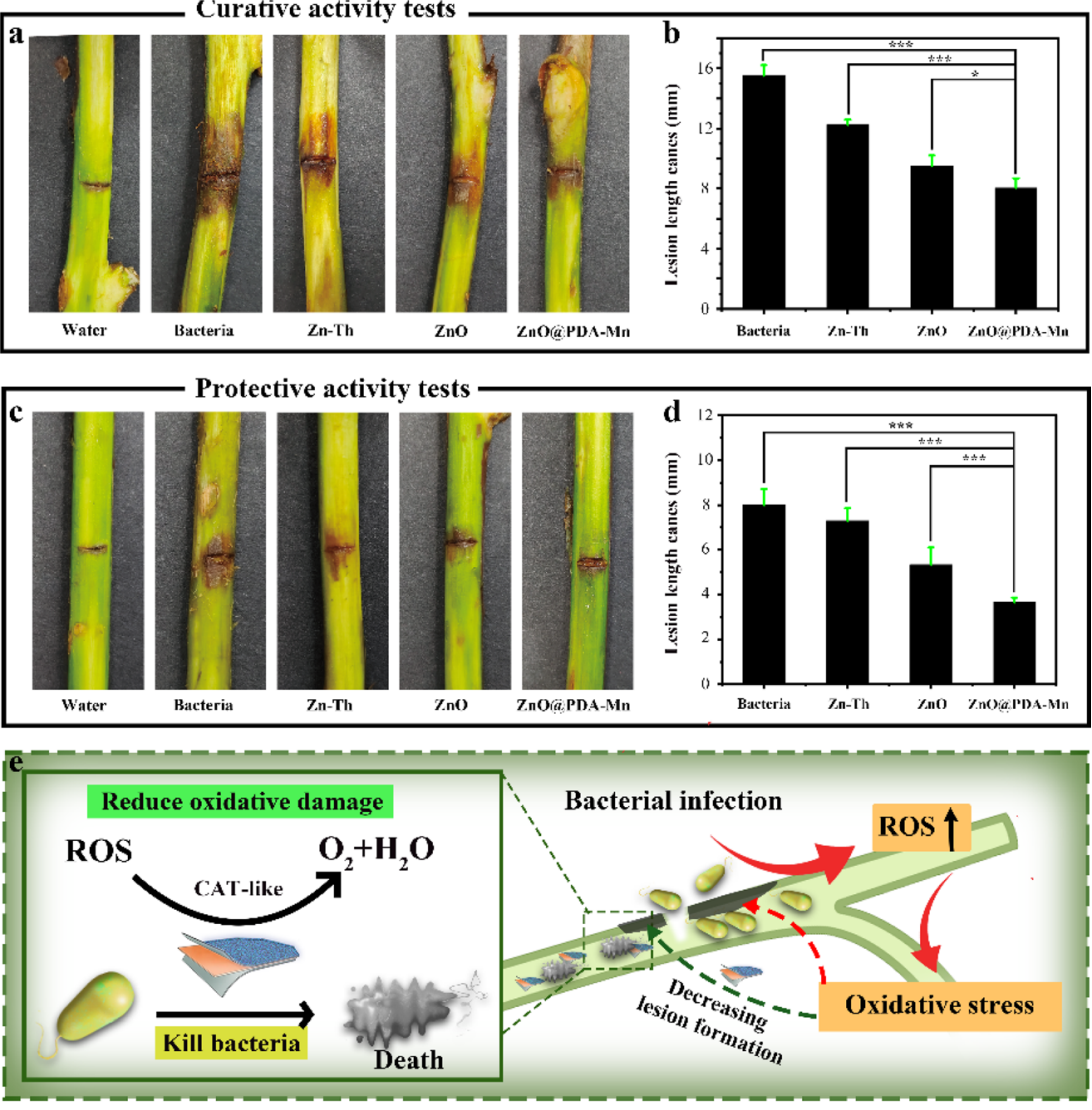
Under controlled greenhouse conditions, the efficacy of Zn–Th, ZnO nanoparticles, and ZnO@PDA-Mn nanocomposites against kiwifruit bacterial canker at a concentration of 200 μg mL^−1^ was assessed. Curative activity (a and b), protective activities (c and d). (e) Schematic diagram of synergistic antibacterial activity of nanocomposites *in vivo*. Significance analysis was carried out by one-way ANOVA and *t* test. Single asterisks indicate *p* < 0.05, double asterisks indicate *p* < 0.01, triple asterisks indicate *p* < 0.001.

**Fig. 7:**
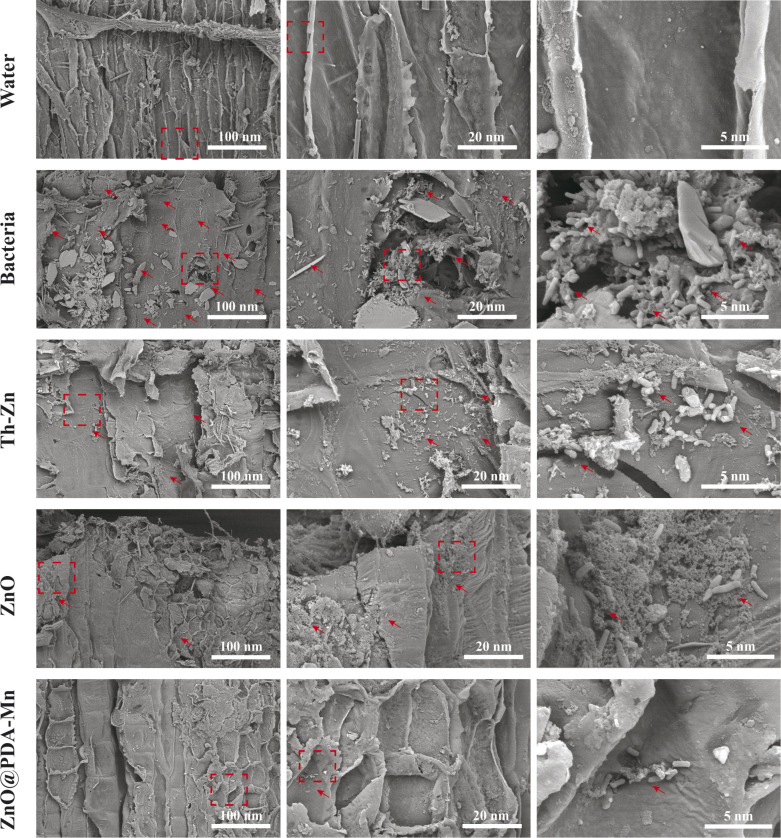
SEM image of kiwifruit tissue section. (The dashed box denotes the zoom position, while the arrow signifies the presence of bacteria).

To investigate the inhibitory effect of ZnO@PDA-Mn on kiwifruit bacterial canker, an *in vivo* experiment was conducted (Fig. 6a–d). The curative experiment was conducted by inoculating *Psa* into healthy kiwifruit branches and treating the kiwifruit with samples 24 h later, and the protection experiment followed the opposite step. Significantly, ZnO@PDA-Mn nanocomposites demonstrated notable curative and protective efficiencies of 47.7% and 53.8%, respectively, at a dosage of 200 μg mL^−1^ against this bacterial infection (Table 1). These effects exceeded those observed with Zn–Th (20.6% and 8.8%) and ZnO (38.7% and 33.8%). During pathogen infection, effector factors can trigger an immune response, leading to the production of ROS.^72^ Elevated ROS levels may exacerbate pathogen invasion.^48^ However, utilizing local ROS, such as H_2_O_2_, effectively can enhance pathogen prevention and control. ZnO@PDA-Mn nanocomposites exhibit high CAT-like catalytic activity, providing a potential means to reduce ROS levels within plant tissues. As illustrated in Fig. 7, there was a conspicuous absence of *Psa* bacteria distribution on the surface of healthy kiwifruit tissue sections. Conversely, a substantial proliferation of *Psa* bacteria was evident on the surface of kiwifruit tissue sections treated with bacteria, resulting in significant damage to the plant tissue. In comparison to kiwifruit treated solely with bacteria, while *Psa* bacteria were present on the surface of kiwifruit tissue sections treated with Zn–Th and ZnO, no discernible pathological impact on plant tissues caused by *Psa* was observed. Notably, only a sparse presence of bacteria was detected on the surface of the kiwi section treated with ZnO@PDA-Mn, elucidating why the kiwi subjected to ZnO@PDA-Mn treatment exhibited minimal lesion development.

**Table 1:** The *in vivo* control efficiency of Zn–Th, ZnO, and ZnO@PDA-Mn against kiwifruit bacterial canker was evaluated at a dose of 200 μg mL^−1^ on kiwi plants. (Different uppercase letters indicate the control efficiencies with a significant difference among different treatment groups at *p* < 0.05)

	Zn–Th	ZnO	ZnO@PDA-Mn
Curative activity	20.6% ± 2.6 A	38.7% ± 4.5 B	47.7% ± 3.9 C
Protective activity	8.8% ± 7.5 A	33.8% ± 8.5 B	53.8% ± 2.5 C

Therefore, the exceptional activity of ZnO@PDA-Mn may be attributed to the high antibacterial activity of the nanocomposites themselves and the reduction in plant ROS levels (Fig. 6e). Through these experimental results, we successfully achieved effective control of bacterial diseases and reduced oxidative damage to plants by leveraging the changes in ROS induced by bacterial infection.

## 4. Conclusions

In summary, we have successfully synthesized multifunctional ZnO@PDA-Mn nanocomposites through a simple and cost-effective method. Our results have shown that the nanocomposites exhibit excellent CAT-like catalytic activity towards efficiently converting H_2_O_2_ into O_2_, thereby reducing ROS levels in under mimic environment. Moreover, the ZnO@PDA-Mn nanocomposites could kill the *Psa* cells *in vitro* through the strong surface interactions with bacteria destruction of bacterial cell membranes. Meanwhile, the addition of H_2_O_2_ did not affect the bactericidal activity of the nanoparticles *in vitro*. Importantly, the CAT-like catalytic activity of the ZnO@PDA-Mn nanocomposites reduced the oxidative damage of plant induced by bacterial infection, and effectively enhanced the control effect of kiwifruit canker disease *in vivo*. Our study has revealed that the combination of active nanoparticles and CAT-like catalytic sites is a simple and effective strategy for highly efficient control the plant bacterial disease. We believe that this novel strategy could also be an effective method to optimize other metal-based nanoparticles for other plant disease, such as plant virus or fungal disease.

## Author contributions

The concept was conceived by S. Y. and L. L.; Z. D. and L. L. designed the experiment and wrote the manuscript. Z. D. was responsible for the preparation, characterization, and performance evaluation of nanoparticles, along with conducting interaction tests between nanoparticles and bacteria or kiwifruit plants. Z. D., Q. S., G. W., and Z. Z. conducted antibacterial activity tests both *in vitro* and *in vivo*. Additionally, Z. Z., G. Z., H. L., Y. C. and X. Z. contributed to data collection for fluorescence imaging of bacteria. S. Y. and L. L. contributed significantly to framing the logical relationships between experimental results and conclusions, as well as refining the manuscript's language. S. Y. supervised the research and revised the manuscript.

## Conflicts of interest

The authors confirm that they have no known competing financial interests or personal relationships that could have influenced the findings presented in this paper.

## Supplementary Material

RA-014-D4RA02006E-s001
