# Supplementary material for: Synthesis of organic–inorganic hybrid nanocomposites modified by catalase-like catalytic sites for the controlling of kiwifruit bacterial canker
Source: RSC Adv. 2024 May 31;14(25):17571–82. doi: 10.1039/d4ra02006e (PMC11140456; doi:10.1039/d4ra02006e)
Supplement: RA-014-D4RA02006E-s001 [file RA-014-D4RA02006E-s001.pdf]

Electronic Supplementary Material (ESI) for RSC Advances.
This journal is © The Royal Society of Chemistry 2024

Supplementary Information

Synthesis of Organic-Inorganic Hybrid Nanocomposites Modified by Catalase-like Catalytic Sites for the Controlling of Kiwifruit Bacterial Canker

Zhenghao Ding, Qingqing Song , Guangdi Wang, Zhuojun Zhong, Guoyong Zhong, Hong Li, Yuexin Chen, Xiang Zhou, Liwei Liu* and Song Yang*

State Key Laboratory of Green Pesticide；Key Laboratory of Green Pesticide and Agricultural Bioengineering, Ministry of Education, Center for R&D of Fine Chemicals of Guizhou University, Guiyang, 550025, China.
* Corresponding author: E–mail: syang@gzu.edu.cn, jhzx.msm@gmail.com.

Supplementary Experimental Section

Characterizations

The morphology of the samples was observed by scanning electron microscopy (SEM; Nova NanoSEM^{TM} 450, FEI, USA) and transmission electron microscopy (TEM; Talos F200C, FEI, USA), and elemental analysis was performed by using an energy dispersive spectroscopy (EDS, Oxford Instruments, X-MAXn65 T, U.K.). We also characterized the structure and composition of the samples by X-ray photoelectron spectroscopy (XPS, ESCALAB 250Xi, Thermo Scientific, USA). All electron spin resonance (ESR) measurements were carried out using a Bruker EMX ESR spectrometer (Billerica, MA) at ambient temperature. Fourier Transform infrared spectroscopy (FT-IR) were recorded in the range of 4000 – 400 cm^{-1} with 32 scans at a resolution of 2 cm^{-1} using FT-IR spectrometer ((Nicolet IS50, ThermoFisher, USA) at room temperature. The zeta potential and size distribution of the samples were determined through a laser diffraction particle size analyzer (DLS, Nano Brook Omni, USA).

Peroxidase (POD)-like activity of nanocomposites.

Steady-state kinetics assays were conducted at 25 °C in 200 μL of phosphate buffer saline solution (PBS, pH 4.5) with 20 μL of ZnO@PDA-Mn nanocomposites solution (final concentration 10 μg/mL) as catalyst in the presence of H_2O_2 and 3, 3', 5, 5'-tetramethylbenzidine (TMB). 1) H_2O_2 as the substrate: 5 μL of 0.0416 M TMB and different amounts of H_2O_2 (5 μL of 0.12%,5 μL of 0.24%, 5 μL of 0.47%,5 μL of 0.94%,5 μL of 1.88%,5 μL of 3.75%,5 μL of 7.5%, 5 μL of 15% H_2O_2) were added to the reaction system containing ZnO@PDA-Mn nanocomposites, with change in absorbance recorded at 652 nm with time at 25°C. 2) TMB as the substrate: 5μL of 15% H_2O_2 and different amounts of TMB (5 μL of 0.0052 M, 5 μL of 0.0104 M, 5μL of 0.0208 M, 5, 10, 15, 20, 25 μL of 0.0416M of TMB solution) were added to the reaction system containing ZnO@PDA-Mn nanocomposite, with the change in

absorbance recorded at 652 nm with time at 25 °C. For comparison, the enzyme-like activities of PDA, ZnO NPs and ZnO@PDA nanocomposites were also measured.

The apparent kinetic parameters were calculated based on Michaelis equation (10.1016/j.biomaterials.2010.11.004).

$$v=V_{max} \times [S]/(K_m+[S])$$

v is the initial velocity, V_{max} is the maximal reaction velocity, [S] is the concentration of substrate and K_m is the Michaelis constant.

Superoxide dismutase (SOD)-like activity of nanocomposites.

The SOD-like activity of the ZnO@PDA-Mn nanocomposites was evaluated at room temperature using a commercial colorimetric SOD assay kit from Solarbio, in accordance with the manufacturer's instructions. The SOD-like activity of ZnO@PDA-Mn (10 μg/mL and 15 μg/mL) was expressed as the percentage inhibition of the competitive WST-1 reaction with superoxide by natural SOD enzymes.

Determination of Hydroxy radical (•OH).

·OH measurement. Glass capillary tubes containing ZnO@PDA-Mn (0.1 mg/mL, 50 μL), H_2O_2 (0.001 M, 50 μL), 5-tert-butoxycarbonyl 5-methyl-1-pyrroline-N-oxide (BMPO, 10mM), and PBS (pH 4.5) were inserted into the electron spin resonance (ESR) cavity to record ·OH signals at selected times.

The interaction between nanoparticles and bacterial surfaces was observed by TEM.

Approximately 5 mL of the bacterial suspension (1×10^9 CFU/mL) was centrifuged at 6000 rpm at 4 ℃ for 1.5 min. After discarding the supernatant, the bacteria were washed with 1 mL PBS thrice to remove the medium; finally, 1 mL PBS was added to resuspend the bacterial solution (5×10^9 CFU/mL). In addition, 100 μL of the bacterial solution was mixed with 5 mL of the samples solution and incubated at 28 ℃ for 4 h. Thereafter, 20 μL aliquots were dropped on a carbon film coated with a copper grid (200 mesh), dried with filter paper for 1 min, and stained with uranium acetate (1%)

for 10 s. The excess uranium acetate was absorbed using a filter paper, and the samples were dried at room temperature, following which they were observed and appropriate images recorded using TEM.

Live/dead cell staining

Bacterial suspensions treated by H_2O_2, ZnO, ZnO@PDA-Mn and ZnO@PDA-Mn $+H_2O_2$ for 4 h. Then the bacterial suspensions were collected by centrifugation at 4000 r/min for 5 min. The cells were then stained with SYTO 9 and propidium iodide (PI) for 30 min and visualized via fluorescence microscopy (FluoroMax®–4P, HORIBA, France).

Zone of inhibition (ZOI) assay

ZnO@PDA-Mn were studied for the antibacterial efficacy against *Psa* using disk diffusion technique commonly known as Kirby Baurer method. This is a qualitative technique to test the ability of a material to inhibit microbial growth by measuring ZOI. Overnight bacterial culture was grown at 37 °C and its cell density was adjusted to 10^8 CFU/mL (i.e. Optical density of 0.1 at 600 nm). 0.1 mL of bacterial culture was spread onto sterile agar plates. Plates were allowed to dried followed by placing 6 mm sterile disc soaked for 10 min in Zn-Th, ZnO and ZnO@PDA-Mn concentration 3.13 μg/mL. Post incubation at 37 °C for 48 h, ZOI around the disc was measured.

Kiwifruit tissue section observation experiment

After measuring the length of kiwifruit lesions, the kiwifruit tissues treated with different samples were sliced. The tissue sections were then immersed in 2.5% glutaraldehyde (Sigma-Aldrich, USA) and fixed for 10 hours at 4 °C under dark conditions. Subsequently, the tissue sections underwent dehydration with a series of ethanol concentrations (30%, 50%, 70%, 90%, and 100%) for 10 minutes each. Finally, the dried tissue sections were sputter-coated with gold for imaging using scanning electron microscopy.

Table S1. Comparison of Michaelis-Menton constants (K_m) and maximum reaction rates (V_{max}) of reported catalase-like nanozymes.

Catalysts	K_m (mM)	V_{max}(mg/(L·min))	Ref.
ZnO@PDA-Mn	116.3	6.03	This work
Catalase	249.4	5.76	10.1016/j.cej.2020.124249
Fe^{3+}/nucleotide CPs	112.2	4.61	10.1016/j.cej.2020.124249
RuO_2 NP	400.0	/	10.1002/chem.201200643
Pt-Ft	420.6	1.6×10^3	10.1016/j.biomaterials.2010.11.004
Au-Si-ACD	117.0	0.23	10.1002/smll.201603051
N-PCNSs-3	66.3	1.03	10.1038/s41467-017-02317-2
IrNCs	132.0	/	10.1016/j.biomaterials.2018.07.049
CN	393.3	4.76	10.1021/acs.nanolett.9b01333
$Au_{24}Ag_1$	222.4	2.21	10.1021/acs.nanolett.0c05148
pero-nanozysome	90.0	4.67	10.1002/adfm.202007130
IrO_x NPs	188.0	10.82	10.1002/anie.201916142

Table S2. Abbreviations

Abbreviation	Full name
Xac	*Xanthomonas axonopodis* pv.*citr*
Psa	*Pseudomonas syringae* pv. *actinidiae*
OD	Optical density
CFU	Colony Forming Unit
POD	Peroxidase
CAT	Catalase
SOD	Superoxide dismutase
PBS	phosphate buffer saline solution
h	Hour
TMB	3,3',5,5'-Tetramethylbenzidine
min	Minute
TEM	Transmission electron microscopy
SEM	Scanning electron microscopy
FT-IR	Fourier Transform infrared spectroscopy
XPS	X-Ray Photoelectron Spectroscopy
XRD	X-Ray Diffraction
DLS	Dynamic light scattering

EDS	Energy dispersive spectroscopy
ROS	Reactive Oxygen Species
PI	Propidium iodide
Zn-Th	Zinc Thiozole
PDA	Polydopamine

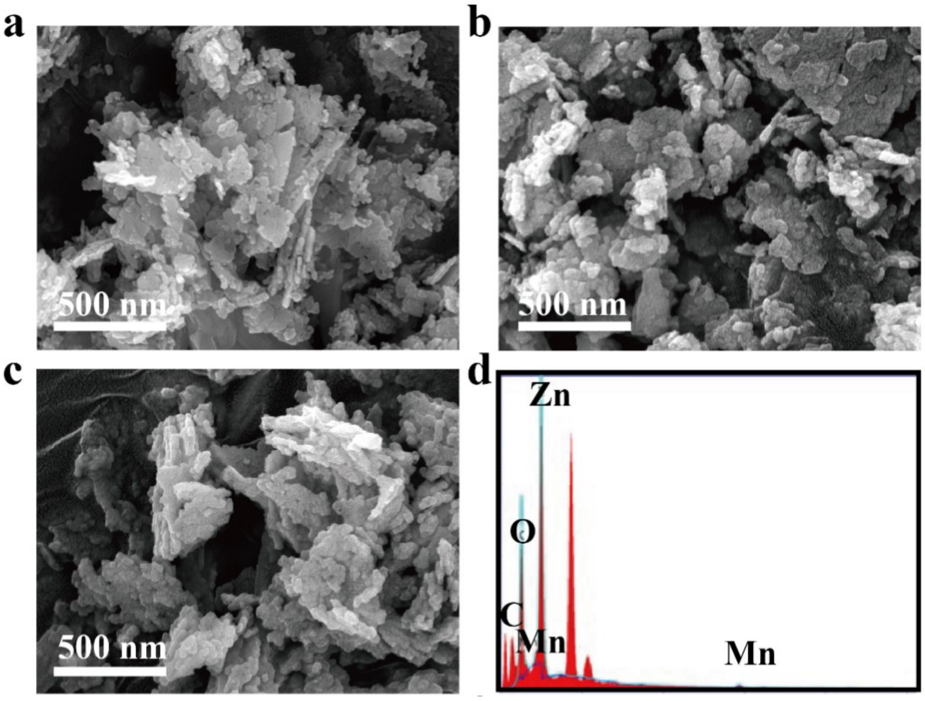

Figure S1. SEM and EDS characterization of (a)ZnO, (b)ZnO@PDA and (c,d)ZnO@PDA-Mn.

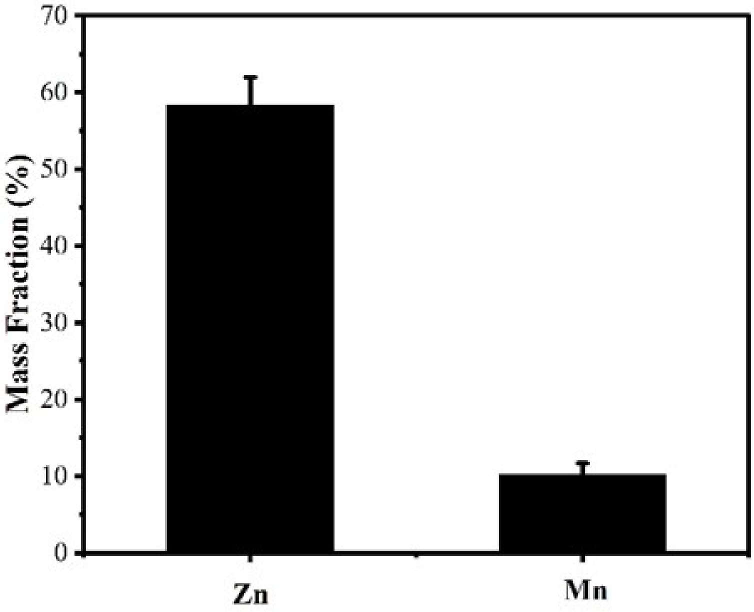

Figure S2. The Mass Fraction of Mn and Zn in ZnO@PDA-Mn.

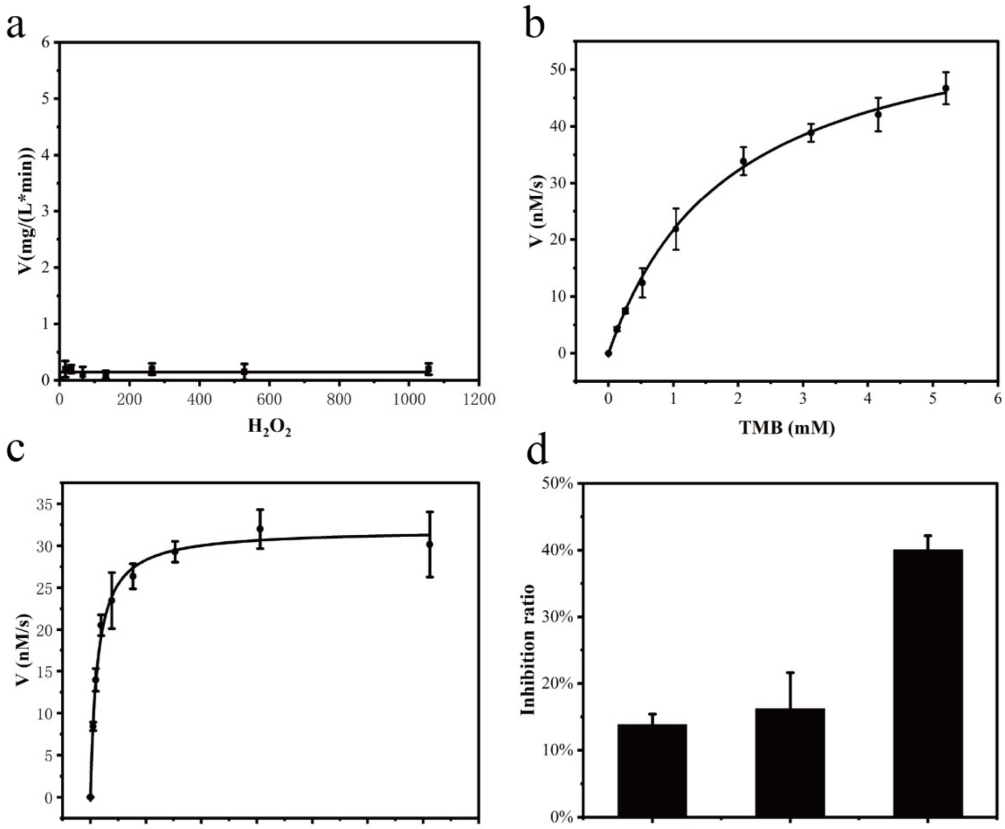

Figure S3. (a) Steady-state kinetics assay of CAT-like activity of PDA with varied H_2O_2. (b) Evaluation of the steady-state kinetics assay for the POD-like activity of PDA using varying concentrations of TMB. (c) Assessment of the steady-state kinetics assay for the POD-like activity of PDA using varying concentrations of H_2O_2. (d) Detection of SOD-like activity of PDA through WST-1 assay.

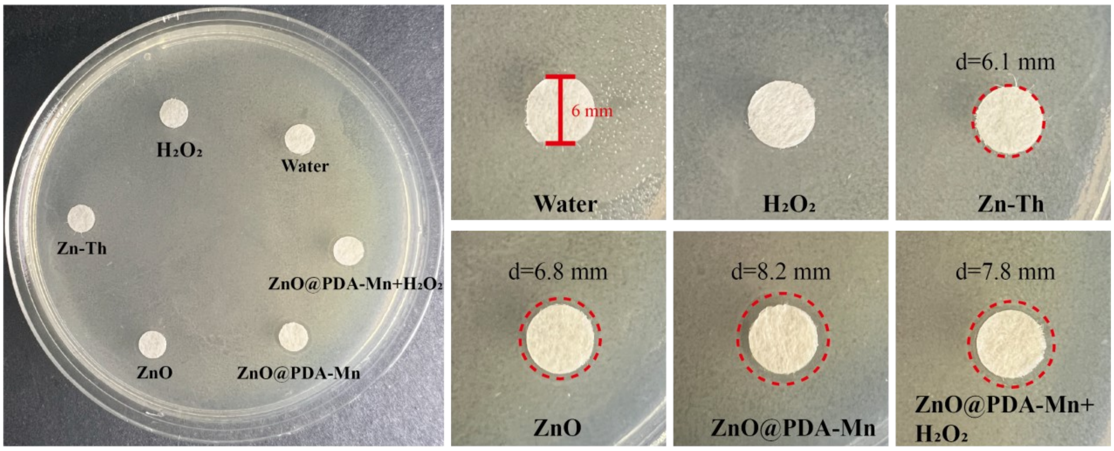

Figure S4. zone of inhibition assay.

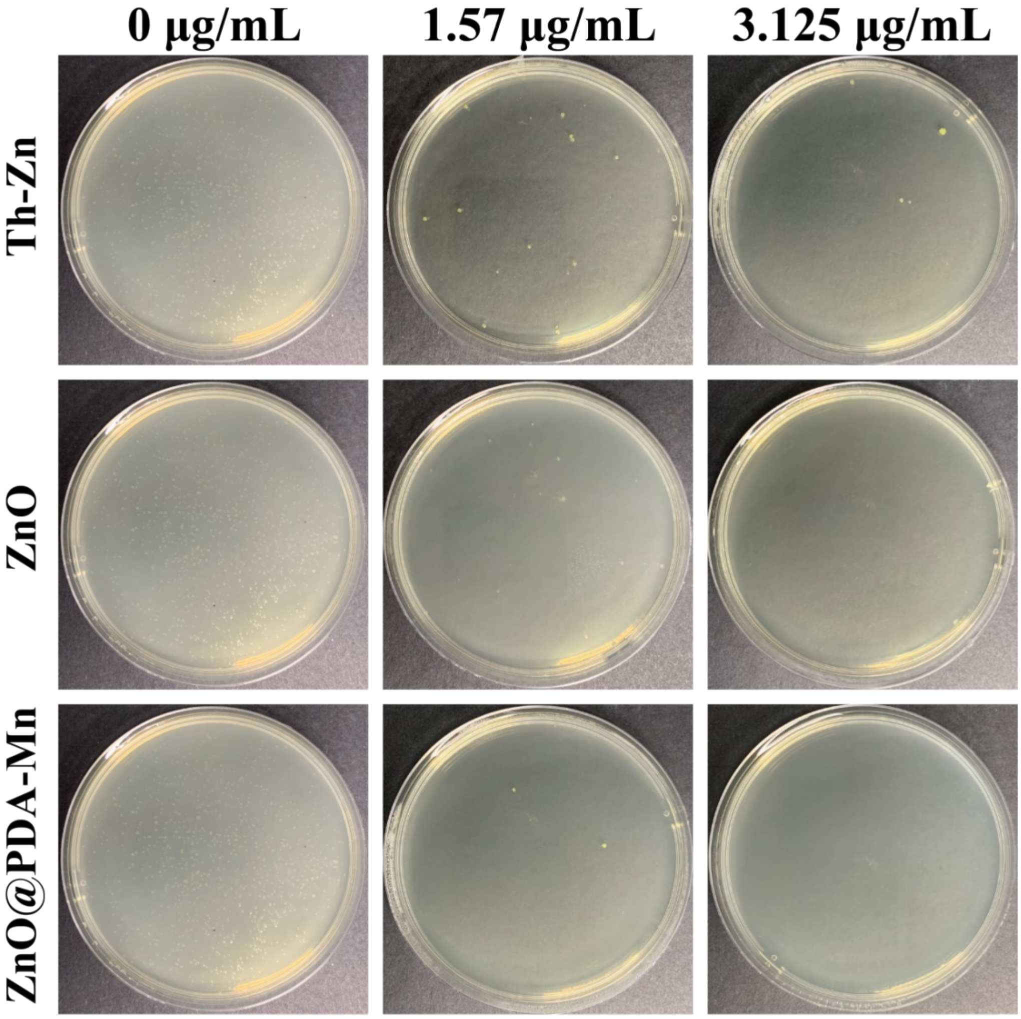

Figure S5. Representative colony formation of *Xanthomonas axonopodis* pv. *citr* (*Xac*) bacteria in different treatment groups.
